# Barriers faced by Ugandan university students in seeking medical care and sexual health counselling: a cross-sectional study

**DOI:** 10.1186/1471-2458-12-986

**Published:** 2012-11-16

**Authors:** Andualem Tadesse Boltena, Farhad Ali Khan, Benedict O Asamoah, Anette Agardh

**Affiliations:** 1 Social Medicine and Global Health, Department of Clinical Sciences Malmo, Lund University, Lund, Sweden; 2 Swedish Institute for Communicable Disease Control, Stockholm, Sweden; 3 Centre for Adolescent Health, Murdoch Children’s Research Institute, Royal Children’s Hospital, University of Melbourne, Melbourne, VIC, Australia

**Keywords:** Acceptability, Accessibility, Availability, Unmet medical care need, Sexual health counselling need, Self-rated health, Youth-friendly, Uganda

## Abstract

**Background:**

Meeting the medical and sexual health care needs of young people is crucial for sustainable development. In Uganda, youth are faced with a number of challenges related to accessing medical care and sexual health counselling services. This study sought to investigate the barriers faced by Ugandan university students in seeking medical care and sexual health counselling.

**Methods:**

This study is part of a cross-sectional survey conducted in 2005 among 980 students at Mbarara University of Science and Technology. Data was collected by means of a self-administered 11-page questionnaire. The barriers encountered by respondents in seeking medical care and sexual health counselling were classified into three categories reflecting the acceptability, accessibility, or availability of services.

**Results:**

Two out of five students reported unmet medical care needs, and one out of five reported unmet sexual health counselling needs. Acceptability of services was the main barrier faced by students for seeking medical care (70.4%) as well as for student in need of sexual health counselling (72.2%), regardless of age, gender, self-rated health, and rural/peri-urban or urban residence status. However, barriers differed within the various strata. There was a significant difference (*p*-value 0.01) in barriers faced by students originally from rural versus peri-urban/urban areas in seeking medical care (acceptability: 64.8%/74.5%, accessibility: 22.0% /12.6%, availability 13.2%/12.9%, respectively). Students who reported poor self-rated health encountered barriers in seeking both medical care and sexual health counselling that were significantly different from their other counterparts (*p*-value 0.001 and 0.007 respectively).

**Conclusions:**

Barriers faced by students in seeking medical and sexual health care should be reduced by interventions aimed at boosting confidence in health care services, encouraging young people to seek early treatment, and increasing awareness of where they can turn for services. The availability of medical services should be increased and waiting times and cost reduced for vulnerable groups.

## Background

Globally, the youth segment of the world’s population is estimated to be over 1.2 billion [1] and about 90% of all young people live in low income countries, mainly in sub-Saharan Africa [2]. Youth form the backbone of every economy and so their health and well-being are very crucial in sustaining a nation’s development [3]. Unmet medical care and sexual health counselling needs in that sector must be taken into account when looking at the health needs of the general population. These are challenges that require attention from public health practitioners, researchers, governments, and the global community-at-large [2].

Several factors account for the high unmet needs outlined above. Young people have higher risk for sexual and reproductive health problems compared to adults [4]. Youthful exuberance affects their way of living and consequently they are more likely to require medical and sexual health counselling [5]. A study by Blum found that most deaths among young people are due to behavioural factors. In addition, the same author concluded that ineffective policies and poor health care systems escalated youth mortality [6]. Poor self-rated health status has also been associated with unmet health care needs [7]. The self-rated health status of a person at a given time was found to be a determinant of future self-rated health [8]. Contemporary health care systems deny youth adequate health care services, compared to children and adults [9]. Health care services for youth have usually been combined with health services intended for children or adults that are not youth friendly. As a result, such programs fail to deliver services earmarked for the health care needs of youth [2,6].

The US, Canada, and Australia have made significant efforts to improve medical and sexual health counselling for young people. The strategy to provide youth-friendly health services has been to allocate specialized human resources and infrastructure. Nevertheless, inequities still exist in seeking health care among youth. In the UK, where not much emphasis has been put on the special needs of young people, they face several barriers in seeking health care, resulting in greater unmet health care and social needs for that population [10].

A Nicaraguan study found that sexual health counselling was sought by only 10% of the users of sexual and reproductive health services in that country [11]. Hesketh and colleagues have suggested that barriers faced by youth in seeking medical care and sexual health counselling are similar all over the world [12]. A study performed among female adolescent youth in the US found that the main barriers to those seeking health care were cost, transportation, lack of respect, and fear of and judgmental attitudes by providers [13]. Another study, done in Pakistan, found long waiting times, unaffordable costs, and negative attitudes on the part of providers as barriers to youth seeking health care [14]. In Sri Lanka, poor availability and limited access to existing health services, a dearth of reproductive health information, lack of confidentiality, and unfriendly attitudes have been identified as major barriers to youth seeking health care [15].

In sub-Saharan Africa countries, finding medical care and sexual health counselling by youth is very challenging. In a study conducted in Ethiopia, the gender of the service provider, the youth friendliness of the health services, and embarrassment over having a sexual health problem were the main barriers to youth in need of sexual health counselling [16]. In Tanzania, the extent to which medical care and sexual health counselling is sought by youth is very poor. Young people in that country appear to have very poor access to sexual and reproductive health and HIV/AIDS services. This is marked by the absence of youth-friendly health services and the hostile tendencies of health service providers [17]. In addition, there are legal and ethical issues confronting providers who attempt to address the health care needs of youth [9]. Moreover, the Tanzanian government decided to leave establishing youth-friendly health services to NGOs, who have been unsuccessful in making them available to the public [17].

In Uganda, the challenges for youth seeking medical care and sexual health counselling are not very different from those in other African nations. The problematic relationship between the health care system and those at the grassroots level, poorly formulated policies, and an inefficient youth health services delivery programs have been the primary factors associated with barriers to seeking medical and sexual health counselling in Uganda [18].

A study conducted in Uganda and Zambia revealed the efficacy of dealing with contextual factors in order to eliminate barriers to sexual health counselling. These factors included culture and its impact on attitudes towards sexuality; the accessibility of sexual and reproductive health services for youth; and the role of care providers [19]. Another study that focused on university students in Uganda identified age, sex, the head of the household’s level of education, and place of origin as socio-demographic factors influencing sexual health among youth [20]. However, no study has been found that has investigated the barriers faced by Ugandan youth in seeking medical and sexual health care.

This study seeks to investigate unmet medical care and sexual health counselling needs, and barriers to obtaining such care that university students in Uganda experience. It is unique in dividing the search for health care into medical versus sexual health counselling. Unmet needs and barriers encountered in seeking medical care or sexual health counselling differ according to the context and must therefore be investigated with regards to socio-demographic characteristics such as sex, age, area of origin, and educational level of the head of the household, as well as self-rated health. Those barriers may be grouped into three categories: availability, accessibility, and acceptability of health care services [5,21,22]. Availability of health care services is defined both as a treatment that is not delivered at a time convenient for the consumer, and as the unavailability of professional help in the area and at the time of need. As a result, individuals experience waiting time as too long and may fail to see a doctor. Problems of cost, transportation, and competing responsibilities are linked to accessibility of health care services. Accessibility issues are commonly associated with people not having sufficient money. Acceptability of health care services refers to personal attitudes toward illness, health care providers, and the health care system [5,21]. Responses associated with acceptability factors are: disappearance of the health problem, perception of not getting any help, respondents claim of not knowing about any good doctor, not having time, wanting to wait for a while (for medical care only), and not feeling confident with existing counselling services (for sexual health counselling only). The aim of this study is to investigate unmet medical care and sexual health counselling need, and barriers to care seeking among university students in Mbarara, Uganda.

## Methods

### Study design, setting, and population

This is a cross-sectional study that used data from a large survey conducted in 2005 among students in Mbarara University of Science and Technology (MUST), Uganda. MUST is a public university established in 1989 and located in the city of Mbarara in southwestern Uganda. Our target population consisted of undergraduate students from the university’s three faculties: medicine, science, and development studies. The sample comprised 1220 students, the entire undergraduate class of MUST in 2005.

Data collection was done by a self-administered 11-page survey containing 132 questions that was drafted by the researchers and modified after discussions with student representatives. Special consideration was given to personal questions. The questionnaire was distributed in lecture halls to 1220 students and had an 80% (980) response rate. The research team monitored the process to ensure confidentiality of the responses. Participants were orally informed about the purpose of the questionnaire and given clear guidelines for filling it out. Written informed consent was collected from all participants, and they were informed they were free to withdraw from the study at any time for any reason. Contact details for the principal investigator and a research assistant were provided in case a participant had questions or concerns that arose due to the questionnaire.

The main variables in this study are socio-demographic factors, self-rated health, unmet medical needs, unmet sexual health counselling needs, and barriers encountered by those seeking medical care or sexual health counselling (availability, accessibility, and acceptability of services). The research project was approved by the Institutional Ethical Review Committee at MUST (DOS 1/6).

### Background variables

The socio-demographic factors used in these analyses were age, sex, place of origin, and the head of household’s level of education. Age was divided arbitrarily into two groups at the upper tertile: “younger” < 24 years old and “older” ≥ 24 years old. Sex was categorised as male or female. Educational level of head of household was categorised into those who completed elementary school and those who did not. Place of origin was categorised into urban/peri-urban versus rural.

Self-rated health, an indicator of how participants classified their general state of current health, was rated on a five-level response scale (*very good, good, fair, bad,* or *very bad*), then dichotomised as follows: *“very good,” “good,”* and *“fair”* were reclassified as *good,* while *“bad”* and *“very bad”* were reclassified as *poor*.

### Dependent variables

Unmet medical care need (UMCN) referred to those participants who, during the three months prior to the survey, experienced a health problem and thought they needed to visit a doctor but abstained from seeking health care. In this variable there were three categories from the original questionnaire: “*yes, several times”*, *“yes”* and “*no.”* For the analysis, “*yes, several times”* and “*yes”* were combined into one category: *“yes”*. The second category of this variable is “*no.”*

The focus of our study was not particularly on whether the students accessed medical care and sexual health counselling in their own areas of residence or through the university medical services but rather whether they had general access to medical care and sexual health counselling services while in school. That not said the questionnaire for this study was administered at the time when the students had been in school for close to 4 months, implying that the previous three months referred to in the questionnaire was a period that the students were generally expected to use university health facilities.

Unmet sexual health counselling need (USCN) corresponded to those who, in the same period, experienced a sexual health problem, thought that they should visit a counsellor, but also abstained from seeking health care. This variable contained the same categories as unmet medical care need.

Barriers experienced in seeking medical care and sexual health counselling referred to reasons given by students who failed to obtain medical care and sexual health counselling when they needed it. Barriers faced in seeking medical care and sexual health counselling were grouped into three categories: acceptability, accessibility, and availability of health care services, based on the responses given by participants.

*Availability* of health care services is defined as a treatment that is not delivered at the convenient time for the consumer; and unavailability of professional help in the area and at the time of need. The responses included are; *waiting time was too long* and *did not succeed to get in touch with a doctor.*

*Accessibility*: This category contained responses related to individual accessibility problems such as cost. The response included in this category is; *did not have enough money*.

*Acceptability*: Acceptability of health care services refers to personal attitude towards illness, health care providers and health care system. This category included the following responses; *the health problem disappeared, do not think I can get any help, did not know about any good doctor, did not have time, wanted to wait for a while (for only UMCN), did not feel confident with existing counselling services (for only USCN)* and *other reason*s.

The definitions and responses used to categorize barriers experienced in seeking medical care and sexual health counselling are from an instrument that has been validated by previously published studies [5,21,22].

### Data analysis

The sample size was established since we assessed all the students at the university, but a formal check revealed that in most analyses a 75% increase of risk could be ascertained at 80% probability. This could not exclude the risk of being unable to detect some true effects of moderate size. The statistical analyses were done with SPSS version 18. A descriptive analysis of frequencies and percentages was performed to describe the population and the factors related to the research question. A cross-tabular descriptive statistical analysis was also performed to analyze the association between predictor and outcome variables. Pearson’s chi square test with a significance level of 0.05 was performed to investigate the association between the different variables.

## Results

Table 1 describes the socio-demographic factors, self-rated health, UMCN, USCN, and barriers faced in care seeking among MUST students. About 43.7% of the students came from rural areas and the rest (56.3%) came from urban and peri-urban areas. The majority of the student population (74.5%) came from families with a household head who had attained high educational level. About 14.9% of the respondents (one in five women and one in ten men) reported poor self-rated health. Two out of five students reported UMCN, while one out of five reported USCN. More women than men reported having experienced UMCN, while the inverse was true for USCN. In all, 70.4% of the students with UMCN rated acceptability of health care services as the major barrier experienced in seeking care, followed by accessibility (16.6%) and availability of health care services (13.0%). For those with USCN, the greatest barrier faced in seeking care was acceptability (72.2%), followed by availability (16.3%), and accessibility of health care services (11.5%).

**Table 1:** Prevalence of socio-demographic factors, self-rated health, unmet medical care and sexual health counseling needs, and barriers faced in seeking care among 980 university students in Uganda

	**All**		**Female**		**Male**	
	**n**	**%**	**n**	**%**	**n**	**%**
*Sex*						
Female	347	35.4				
Male	633	64.6				
*Age*						
Younger (< 24)	628	65.6	250	75.1	378	60.6
Older (≥ 24)	329	34.4	83	24.9	246	39.4
Missing	(23)		(14)		(9)	
*Area of origin*						
Rural	424	43.7	106	31.0	318	50.6
Urban/peri-urban	546	56.3	236	69.0	310	49.4
Missing	(10)					
*Educational level of head of household*						
Post Primary school	688	74.5	274	84.8	414	69.0
≤ Primary school	235	25.5	49	15.2	186	31.0
Missing	(57)					
*Self-rated health*						
Good	730	85.1	254	83.6	476	85.9
Poor	128	14.9	50	16.4	78	14.1
Missing	(122)					
*Unmet medical care needs*						
Yes	377	41.0	140	43.1	237	39.8
No	543	59.0	185	56.9	358	60.2
Missing	(60)					
*Unmet sexual health counselling needs*						
Yes	182	20.8	54	17.5	128	22.7
No	691	79.2	254	82.5	437	77.3
Missing	(107)					
*Barriers to medical care seeking*						
Acceptability	369	70.4	128	71.5	241	69.9
The health problem disappeared	138		49		89	
Did not think I can get any help	29		5		24	
Did not know about any good doctor	12		3		9	
Did not have time	24		8		16	
Wanted to wait for a while	70		34		36	
Other reasons	96		29		67	
Accessibility	87	16.6	27	15.1	60	17.4
Did not have enough money	87		27		60	
Availability	68	13.0	24	13.4	44	12.8
Waiting time was too long	33		14		19	
Did not succeed to get in touch with a doctor	35		10		25	
Missing	(48)		20		28	
*Barriers to sexual health counselling seeking*						
Acceptability	244	72.2	69	69.0	175	73.5
The health problem disappeared	105		27		78	
Did not think I can get any help	34		6		28	
Did not know about any good counsellor	38		9		29	
Did not feel confident with existing counselling services	67		27		40	
Accessibility	39	11.5	15	15.0	24	10.1
Did not have enough money	39		15		24	
Availability	55	16.3	16	16.0	39	16.4
Waiting time was too long	27		8		19	
Did not succeed to get in touch with a counsellor	28		8		20	
Missing	(10)		1		9	

Table 2 describes cross-tabular associations of UMCN and USCN with socio-demographic factors and self-rated health. About 40% of students below age 24 reported UMCN. Slightly more rural students (43.3%) reported UMCN, compared to urban/peri-urban students (40%). Students that had heads of family whose educational level was below elementary school reported slightly more UMCN (43%) than those with family heads whose education went beyond the elementary school level (40%). Three- quarters of all students with poor self-rated health status indicated having UMCN.

**Table 2:** Cross-tabular association of unmet medical care and sexual health counseling needs with socio-demographic factors and self-rated health among university students in Uganda

	**All**		**Unmet medical care need**		**All**		**Unmet sexual health counselling need**	
	**n**	**%**	**n**	**%**	**n**	**%**	**n**	**%**
*Sex*								
Female	325	35.3	140	43.1	308	35.3	54	17.5
Male	595	64.7	237	39.8	565	64.7	128	22.7
Missing	(60)				(107)			
*Age*								
Younger < 24	599	66.6	237	39.6	563	66.0	110	19.5
Older ≥ 24	301	33.4	124	41.2	290	34.0	65	22.4
Missing	(80)				(127)			
*Area of origin*								
Rural	395	43.3	171	43.3	376	43.4	79	21.0
Urban/ peri-urban	517	56.7	202	39.1	490	56.6	103	21.0
Missing	(68)				(114)			
*Educational level of head of household*								
≤ Primary	654	74.9	258	39.4	620	74.7	126	20.3
Post Primary school	219	25.1	96	43.8	210	25.3	48	22.9
Missing	(107)				(150)			
*Self-rated health*								
Good	699	84.9	250	35.8	667	85.1	117	17.5
Poor	124	15.1	88	71.0	117	14.9	45	38.5
Missing	(157)		(43)		(196)			

USCN was reported by 18% of the women and 23% of the men. Among students under age 24, 20% reported USCN. The prevalence of USCN was equally distributed among students from rural and urban areas. Students whose heads of families had educational levels beyond elementary school reported slightly more USCN (23%) than those whose heads of family had less than an elementary school education (20%). Nearly 40% of those students with poor self-rated health status also reported having USCN.

Table 3 describes the subgroup analysis of barriers faced in seeking medical care by students who reported UMCN. Among women reporting UMCN, the major barriers to obtaining medical care were acceptability (72%), accessibility (15%), and availability (13%). Men also reported a similar pattern. For them the barriers experienced in seeking medical care were acceptability (70%), accessibility (17%), and availability (13%) of health care services. Among students from rural areas, the major barriers found in seeking medical care were acceptability (65%), accessibility (22%), and availability (13%) of health care services. The results showed a significant difference (*p*-value 0.01) in barriers experienced in seeking care among rural and urban/peri-urban students. Of the students who indicated poor self-rated health 53%, 26%, and 21% showed acceptability, accessibility, and availability, respectively, as the major barriers faced in seeking medical care (*p*-value < 0.001).

**Table 3:** Analysis of barriers to medical care seeking among 524 students who reported unmet medical care needs among university students in Uganda

	**All**		**Acceptability**		**Accessibility**		**Availability**		**P-value**
	**n**	**%**	**n**	**%**	**n**	**%**	**n**	**%**	
*Sex*									
Female	179	34.2	128	71.5	27	15.1	24	13.4	0.79
Male	345	65.8	241	69.9	60	17.4	44	12.8	
Missing	(456)	46.5							
*Age*									
Younger < 24	331	65.4	232	70.1	61	18.4	38	11.5	0.16
Older ≥ 24	175	34.6	124	70.9	23	13.1	28	16.0	
Missing	(474)	48.4							
*Area of origin*									
Rural	227	43.6	147	64.8	50	22.0	30	13.2	0.01
Urban/ peri- urban	294	56.4	219	74.5	37	12.6	38	12.9	
Missing	(459)	46.8							
*Educational level of head of household*									
Post Primary school	347	69.7	247	71.2	55	15.9	45	13.0	0.76
≤ Primary school	151	30.3	104	68.9	28	18.5	19	12.6	
Missing	(482)	49.2							
*Self-rated health*									
Good	370	79.7	281	75.9	49	13.2	40	10.8	< 0.001
Poor	94	20.3	50	53.2	24	25.5	20	21.3	
Missing	(516)	(52.7)							

Table 4 shows subgroup analysis of barriers encountered in seeking sexual health counselling by students who reported USCN. Among such women, the major barriers they experienced were acceptability (70%), followed by availability (16%), and accessibility (15%). Similarly, men who reported USCN indicated the barriers they found in seeking sexual health counselling to be acceptability (74%), availability (16%), and accessibility (10%). Among the students under age 24, the major barriers found were acceptability (74%), availability (15%), and accessibility (12%). Students with poor self-rated health (*p*-value 0.007) indicated acceptability (61%) as the greatest barrier encountered in seeking sexual health counselling, followed by availability (29%), and accessibility (11%).

**Table 4:** Analysis of barriers to sexual health counseling seeking among 338 students who reported unmet sexual health counseling needs among University students

	**All**		**Acceptability**		**Accessibility**		**Availability**		**P-value**
	**n**	**%**	**n**	**%**	**n**	**%**	**n**	**%**	
*Sex*									
Female	100	29.6	69	69.9	15	15.0	16	16.0	
Male	238	70.4	175	73.5	24	10.1	39	16.4	0.46
Missing	642	(65.5)							
*Age*									
Younger < 24	198	60.7	146	73.7	23	11.6	29	14.6	
Older ≥ 24	128	39.3	91	71.1	12	9.4	25	19.5	0.43
Missing	(654)	(66.7)							
*Area of origin*									
Rural	152	45.0	102	67.1	21	11.3	23	12.4	
Urban/ peri-urban	186	55.0	142	76.3	18	11.8	32	21.1	0.08
Missing	(642)	(65.5)							
*Educational level of head of household*									
Post Primary school	229	70.9	173	75.5	21	9.2	35	15.3	
≤ Primary school	94	29.1	63	67.0	15	16.0	16	17.0	0.17
Missing	(657)	(67.0)							
*Self-rated health*									
Good	231	78.3	175	75.8	28	12.1	28	12.1	
Poor	64	21.7	39	60.9	7	10.9	18	28.1	0.007
Missing	(685)	(69.9)							

## Discussion

Socio-demographic factors and self-rated health were clearly correlated with UMCN and USCN among university students in Uganda. We found strong statistical associations between UMCN and USCN and self-rated health. USCN was especially prevalent among students under age 24. The study also revealed a statistically significant association between area of origin and perceived barriers to seeking medical care.

UMCN was reported to be more prevalent among young women than men, just as in previous studies in Canada [23] and the US [2]. More men than women had USCN than UMCN. Perhaps this is because most efforts to improve sexual and reproductive health among young people are directed toward the female population.

The major barrier to seeking medical care or sexual health counselling cited by prospective student clients was the acceptability of health care services. The second barrier was accessibility (for UMCN) or availability (for USCN). Similar results were found in the overall student population as among those with poor self-rated health. The differences we found in barriers faced by those seeking medical care and sexual health counselling contrast with a study by Sibley & Glazier [21], which concluded that availability always leads acceptability and accessibility as the primary cause of unmet health care needs. In our study, acceptability of services was consistently found to be the major barrier encountered in seeking health care. It may be that people fear social, financial, or political consequences of seeking medical and sexual counselling services. Since acceptability depends on information and attitudes regarding health care services, attempts to reduce UMCN and USCN should boost confidence in health care services, encourage students to seek early treatment, and promote awareness of where they can go for their medical and sexual health needs.

Our study demonstrated a statistically significant difference (*p*-value 0.01) between area of origin and barriers found in seeking medical care. This stands in strong contrast to Chen & Hou’s 2002 study [22]. We found accessibility to be a stronger barrier to obtaining medical care for rural than urban or peri-urban students. In this study, not seeking medical care due to cost of services was the main indicator used to evaluate accessibility barriers medical care. Therefore it is imperative that financial barriers that intensify the rural–urban gap in UMCN be removed.

We also found that poor self-rated health correlated with UMCN, in agreement with a study that was conducted among those incarcerated for substance abuse [7]. Lower self-rated health was strongly linked to barriers experienced by students seeking medical care. Those with poorer self-rated health had more pronounced challenges with regard to accessibility and availability of health care services compared to those of their peers who reported good self-rated health. This is a crucial issue of health equity that needs to be addressed.

A unique aspect of our study was the classification of unmet health care needs into two distinct categories, UMCN and USCN, in order to reveal similarities and differences. We analyzed the barriers of availability, accessibility, and acceptability experienced by students at a university in sub-Saharan Africa who sought medical care or sexual health counselling. Our findings are supported by previous studies of unmet health care needs [2,7,21-23]. The response rate in our study was high, making it easier to generalize the results.

### Limitations of the study

This study was performed by means of a cross-sectional design, which may present difficulties in ascertaining the direction of causality between the variables analyzed.

UMCN and USCN were restricted to a period of three months to help reduce recall bias. We were unable to determine whether a large segment of the target group might have received better care compared to their counterparts in the same age group. The survey might be vulnerable to reporting bias, response bias, and selection bias. The lack of adjusting for potential confounders is also a limitation.

## Conclusions

We recommend that a) equal counselling and health care provision be made for young men and women on issues of sexual and reproductive health, b) barriers to seeking medical care and sexual counselling services be reduced by intervention aiming at boosting student confidence in health care services, encouraging young people to seek early treatment, and increasing awareness of where they can turn for services, and c) reducing waiting time, lowering cost, and increasing the availability of medical services for vulnerable groups.

## Competing interests

The authors declare that they have no competing interests.

## Authors’ contributions

ATB was involved in the writing and data analysis, FK was involved in the writing and data analysis, BOA was involved in the writing and data analysis and AA was involved in the writing, data analysis and study design. All authors read and approved the final manuscript.

## Pre-publication history

The pre-publication history for this paper can be accessed here:

http://www.biomedcentral.com/1471-2458/12/986/prepub
